# Supplementary material for: “Polymerization” of Bimerons in Quasi-Two-Dimensional Chiral Magnets with Easy-Plane Anisotropy
Source: Nanomaterials (Basel). 2024 Mar 11;14(6):504. doi: 10.3390/nano14060504 (PMC10974324; doi:10.3390/nano14060504)
Supplement: Supplementary file 1 [file nanomaterials-14-00504-s001.zip › Supplementary movies.pdf]

Supplementary videos

Supplementary video S1

Transformation of the (+)roundabout with N = 5 constituent bimerons into a buckled chain, which occurs due to the displacement instability of the central meron with the topological charge Q=+1/2. The central meron performs a circular movement around the ring center with the increasing amplitude until it annihilates by merging with a (Q = −1/2)-anti-meron within the boundary.

Supplementary video S2

Transformation of the (−)roundabout with N = 7 constituent bimerons into a looped chain. In this case, no bimeron annihilation occurs since all merons possess the same topological charges -1/2. The central meron just beats two exterior bimerons out of their shallow energy minimum defined by the interaction potential.

Supplementary video S3

The crossing with N = 5 of exterior bimerons rearranges into a macromolecule, which consists of the (N = 3)-crossing with some parts of chains attached to it.

Supplementary video S4

Disintegration process of a bimeron macromolecule, which combines a (+) roundabout with N = 6 constituent bimerons and two bimerons attached to it and forming two (N=3) crossings.

Supplementary video S5

Transformation of a modulated state with the hexagonal arrangement of (-) and (+) roundabouts into a disordered bimeron polymer.

Supplementary video S6

Transformation of the hexagonal SkL into a disordered bimeron polymer as achieved by changing the uniaxial anisotropy from −0.5 to −1.5.

Supplementary video S7

Transformation of the disordered bimeron polymer into a hexagonal ordering of skyrmions as achieved by changing the uniaxial anisotropy from −1.5 to −0.5.
